# Reaching proficiency in robotic liver surgery in two high-volume European HPB centers

**DOI:** 10.1007/s11701-026-03372-y

**Published:** 2026-04-03

**Authors:** R. W. J. J. van Dorst, I. H. M. Borel Rinkes, S. Gilg, I. Q. Molenaar, J. Hagendoorn, E. Sparrelid

**Affiliations:** 1 Department of Surgical Oncology, University Medical Center Utrecht and Utrecht University, Heidelberglaan 100, 3584CX, Utrecht, The Netherlands; 2 Division of Surgery and Oncology, Department of Clinical Science, Intervention and Technology, Karolinska Institutet, Karolinska University Hospital, Hälsovägen, Huddinge, 13 141 57, Stockholm, Sweden

**Keywords:** Robotic surgery, Liver surgery, Minimally invasive

## Abstract

Robotic liver surgery is increasingly used in hepatobiliary surgery. University Medical Center Utrecht and the Karolinska University Hospital are both tertiary centers that have adopted robotic surgery with distinct implementation strategies. Analysis of these strategies may benefit surgeons starting up a robotic liver surgery program. A natural experiment comparing an unguided early adopter versus a guided majority adopter was performed. Rolling averages per block of 20 patients were used to find the required number of cases to proficiency, defined as < 10% severe complications (Clavien-Dindo ≥3 A) and < 10% conversion rate. 431 patients were included. The moving average analysis shows that the proficiency threshold of < 10% conversion rate and < 10% severe complication rate in a block of 20 cases was reached at patient 124 at UMCU and 133 at KUH. Guidance leads to a safe and rapid expansion of a surgical team with varying experience levels of surgeons. To increase complexity an intentional institutional focus is required with more limited team size. Early definition of institutional goals and alignment of training pathways is vital for efficient and sustainable program development.

## Introduction

Minimally invasive surgery is evolving rapidly. Recently, the increased implementation of robotic surgery offers advantages over conventional laparoscopy, including increased instrumental dexterity, precision, and 3D-visualization [1, 2]. This development has expanded the feasibility of using a minimally invasive approach for complex procedures such as hepato-pancreato-biliary (HPB) surgery [3, 4].

Evaluations suggest that although initial investment of time and means are high, improvement of care can be achieved in high-volume centers through reduced complications, shorter hospital stays, and faster recovery [2, 5, 6]. In oncologic HPB surgery, robot-assisted approach has demonstrated comparable R0 resection rate and lymph node removal to open surgery; while providing reduced blood loss, conversion rates and length of hospital stay, especially in minor and technically complex liver resections [7–10]. Nonetheless, variability in adoption of robotic liver surgery persists across institutions, reflecting uncertainty about sustainability, training pathways, and the organizational strategies needed for successful implementation [4, 7, 11–13].

The University Medical Center Utrecht (UMCU), a tertiary referral center in the Netherlands, started performing robot-assisted HPB surgery in September 2014. At the time, robot-assisted HPB surgery was newly introduced in the Netherlands and UMCU is considered an early adopter, pioneering the use of the robot in procedures of increasing complexity and extending indications for robotic application [1, 2, 4, 14].

The Karolinska University Hospital (KUH) is Sweden’s largest hospital, affiliated with the university Karolinska Institutet, with high-volume specialized HPB surgery. Similarly to the Netherlands, HPB surgery is centralized to university hospitals with regional affiliation. The KUH first introduced robot assistance for HPB surgery in October of 2020 [15]. At this time beginning with robotic HPB surgery represents a majority adopter in Europe [16]. Program implementation was supported through structured collaboration with UMCU, leveraging proctorship and prior institutional experience [15]. The present study aims to describe and evaluate this structured implementation of robotic liver surgery supported by international collaboration and experienced proctors, thereby providing a reproducible framework for broader adoption and an insight into the institutional development process.

It is hypothesized that structured guidance in implementation leads to safe and rapid expansion of the number of surgeons in robot-assisted surgery without delay in reaching institutional proficiency, while rapid progression in complexity is safely possible with a focused team and institutional strategy.

## Methods

### Study design and population

The study was performed as a natural experiment, facilitated by the two different time periods and strategies when respective hospitals started implementing robotic surgery. The patients were allocated to an institutional treatment pathway in the early adopter or the majority adopter clinic, determined by geography and time period.

The cohort is formed of all patients receiving robotic surgery of the liver in the department of HPB surgery at KUH and UMCU. The KUH cohort starts at the advent of implementation of robot-assisted HPB surgery at KUH in October 2020 leading up to and including 2024 when it is deemed that the learning curve will have been completed and multiple surgeons will have been successfully introduced to robotic liver surgery. The period of introduction of robot-assisted surgery at UMCU lasted from 2014 to 2021, due to the lower volume of total liver surgery. These chronological cases were recorded. At UMCU the robotic system used was initially Da Vinci Si (Intuitive Surgical), later replaced by Da Vinci Xi (Intuitive Surgical). At KUH the robotic system used from the outset was Da Vinci Xi (Intuitive Surgical).

Data at KUH was collected prospectively and kept in a safe repository with access limited to members of the study team. All data was kept anonymized with a key available to link to patient files in the electronic patient record at KUH. Data from patients at UMCU was collected retrospectively and stored in a comparable fashion. Permission was granted by respective local institutional review boards. Due to the nature of the study design, informed consent was waived.

### Data collection

General patient characteristics that were collected include: Age, sex, BMI, medical history, previous abdominal surgery, American Society of Anesthesiologists (ASA) score. Pertaining to the disease, number of tumors, final pathological entity, and pre-operative chemotherapy were assessed. Biochemical parameters were CA19.9, AFP or CEA where applicable.

Intra-operative parameters were operative time in minutes, estimated blood loss, conversion to open procedure and reason for conversion, usage of the Pringle maneuver, time under Pringle when applied. Length of stay in days post-operatively, occurrence of complications in the first 30 days, scored according to the Clavien-Dindo classification, with ≥3 A being classified as severe complications, and radicality of resection (R0/R1), were the post-operative outcome measures.

### IWATE Criteria

In order to assess difficulty of resection the IWATE criteria were used [17, 18]. The IWATE criteria pertain to a six parameter scoring system to classify difficulty of laparoscopic hepatectomy in 4 difficulty levels (low, intermediate, advanced and expert). The IWATE criteria have also been validated as predictive of outcome in robotic liver surgery [19–21]. The scoring is based on tumor location, tumor size, proximity to major vessels, extent of resection, presence of hand assisted surgery and pre-operative liver function.

### Defining proficiency

Institutional proficiency was pre-defined as an average conversion rate < 10% and a severe complication rate < 10%, based on the literature and the approach of this comparative analysis [22–25]. These parameters were chosen to reflect institutional safety and successful implementation, rather than individual surgeon skill or learning and set to benchmark thresholds in minimally invasive hepatectomy [24, 26–31]. Secondary outcomes for proficiency were estimated blood loss and operative time.

### Statistical analysis

The data were analyzed using IBM SPSS Statistics (Version 29.0.1.0 or newer). Continuous data was reported as median with interquartile range (IQR). Categorical data was counted with percentages given. Data is split according to center where the robotic liver surgery was performed and described separately and as a single cohort. To compare continuous outcomes the Mann-Whitney U test was used. The Chi-squared test was used for categorical and binary outcomes. In order to show frequency and proportion of IWATE difficulty classification data were stratified by semester and presented as a stacked bar chart. A two-sided p-value of < 0.05 was deemed to be statistically significant.

To evaluate reaching the proficiency threshold, a moving average time-to-proficiency analysis was performed. Moving average blocks of 20 consecutive cases were used to calculate block-wise conversion rate and severe complication rate (Clavien-Dindo ≥ 3 A). The first chronological case in which both moving averages crossed below the threshold was counted as the point of institutional proficiency.

To assess independent influence of variables associated with operative outcomes, regression analysis was performed. Binary logistic regression was used to evaluate predictors of all early complications. The chronological case number was entered as a continuous variable to evaluate change over time. There were adjustments for case complexity using the IWATE difficulty score and the different console surgeons. To elucidate and correct for individual differences in learning curves an interaction factor between chronological case number and console surgeon was added to the model.

Estimated blood loss, operative time and length of postoperative hospital stay were analysed using multivariate regression analyses with clinically relevant covariates, patient characteristics and operative parameters to identify factors associated with secondary outcomes.

## Results

### Patient characteristics and operative parameters

Table 1 displays patient characteristics. 431 patients were included across the total study period, 267 were treated at KUH and 164 at UMCU. Patients at KUH were significantly older than those at UMCU (median 68 vs. 63 years, *p*<.001). While distribution of sex, BMI and most comorbidities were comparable between centres, the patients treated at KUH more frequently had a history of cardiovascular disease (43.1% vs. 18.3%, *p*<.001) and an ASA score ≥ 3 (47.9% vs. 16.2%, *p*<.001), whereas prior abdominal surgery was more common at UMCU (59.1% vs. 40.1, *p*<.001). The distribution of pathological diagnoses differed significantly between centres (*p*<.001), with colorectal liver metastases and hepatocellular carcinoma being more frequent at UMCU, while benign lesions and gallbladder cancer were more commonly prevalent at KUH.

**Table 1:** Patient characteristics

	Total (*N* = 431)	KUH (*N* = 267)	UMCU (*N* = 164)	*P*-value
Age (years)	67(19)	68(18)	63.0(22)	< 0.001
Female sex	230(53.4)	148(55.4)	82(50.0)	0.273
BMI (kg/m^2^)	25.7(6.1)	25.8(5.7)	25.3(6.4)	0.432
ASA score ≥ 3	171(39.7)	128(47.9)	43(16.2)	< 0.001
Prior abdominal surgery	204(47.3)	107(40.1)	97(59.1)	< 0.001
Diabetes	77(17.9)	50(18.7)	27(16.5)	0.552
Hypertension	177(41.1)	113(42.3)	64(39.0)	0.499
Cardiovascular Disease	145(33.6)	115(43.1)	30(18.3)	< 0.001
Renal comorbidity	41(9.5)	30(11.2)	11(6.7)	0.120
Pulmonary disease	104(24.1)	69(25.8)	35(21.3)	0.289
Anticoagulant use	77(17.9)	53(19.9)	24(14.6)	0.170
Cirrhosis Child-Pugh A	35(8.1)	23(8.6)	12(7.3)	0.476
Cirrhosis Child-Pugh B	2(0.5)	2(0.7)	0	0.476
> 1 lesions	94(21.8)	48(18.0)	46(28.0)	0.014
Neo-adjuvant chemotherapy	66(13.3)	44(16.5)	22(13.4)	0.391
**Diagnosis in pathology**				< 0.001
Colorectal metastasis	154(35.7)	73(27.3)	81(49.4)	
Hepatocellular carcinoma	71(16.5)	41(15.4)	30(18.3)	
Gall bladder cancer	22(5.1)	21(7.9)	1(0.6)	
Non-colorectal metastasis	23(5.3)	12(4.5)	11(6.7)	
Cholangiocarcinoma	18(4.2)	12(4.5)	6(3.7)	
Other	8(1.9)	8(3.0)	0	
Benign	135(31.3)	100(37.5)	35(21.3)	
**Tumour markers**				
CA19.9 (U/ml)	12(21.4)	13(21.3)	3.9(20.9)	0.326
AFP (ng/ml)	4.2(8.5)	4.5(7.4)	3.9(11.6)	0.916
CEA (ng/ml)	2.9(5.2)	2.6(2.6)	4.9(12.9)	0.002

Values are expressed as frequencies (percentage) or median (IQR). BMI, body mass index; ASA, American Society of Anesthesiologists; KUH: CA19.9(*N* = 115), AFP(*N* = 55), CEA(*N* = 119); UMCU: CA19.9(*N* = 7), AFP(*N* = 30), CEA(*N* = 43)

Regarding the operative parameters shown in Table 2, conversion to an open procedure was more common at KUH (11.2% vs. 4.3%, *p*=.012). Reason for conversion did not differ significantly between centres. Operative time was similar between centres, estimated blood loss was greater at KUH (median 100 vs. 50 ml, *p*<.001). The Pringle manoeuvre was applied more frequently at KUH (66.7% vs. 40.9, *p*<.001), although when applied, median Pringle time was longer at UMCU (30 vs. 23 min, *p*<.001). Rate of major post-operative complications did not differ significantly between the two hospitals. Post-operative length of stay was shorter at KUH (median 3 vs. 4 days, *p*<.001). Rates of R0 resection were generally high, but significantly higher at UMCU compared to KUH (92.7% vs. 82.4%, *p*=.003). There was a significant difference in the IWATE difficulty scores between the two hospitals in the cases performed. Half of the cases at KUH were low difficulty, while this only represented a quarter of cases at UMCU. With 23 (14.4%) of cases at expert level at UMCU, this was significantly more than 6 (2.2%) at KUH.

**Table 2:** Operative parameters

	Total (*N* = 431)	KUH (*N* = 267)	UMCU (*N* = 164)	*P*-value
Conversion to open procedure	37(8.6)	30(11.2)	7(4.3)	0.012
Reactive conversion	8(21.6)	6(20.0)	2(28.6)	0.620
Proactive conversion	29(78.4)	24(80.0)	5(71.4)	0.620
EBL (ml)	50(180)	100(160)	50(195)	< 0.001
Operative time (minutes)	150(106)	154(104)	147.5(110)	0.215
Pringle manoeuvre	245(56.8)	178(66.7)	67(40.9)	< 0.001
Pringle time (minutes)	25(20)	23(15)	30(15)	< 0.001
Clavien-Dindo ≥3 A	34(7.9)	18(6.7)	16(9.8)	0.260
Post-operative Length of stay (days)	3(3)	3(2)	4(3)	< 0.001
R0-resection	372(86.3)	220(82.4)	152(92.7)	0.003
**IWATE Difficulty Score**				< 0.001
Low	173(40.1)	134(50.2)	39(24.4)	
Intermediate	172(39.9)	89(33.3)	83(51.9)	
Advanced	53(12.3)	38(14.2)	15(9.4)	
Expert	29(6.7)	6(2.2)	23(14.4)	

Values are expressed as frequencies (percentage) or median (IQR)

### Reaching institutional proficiency

**Fig. 1:**
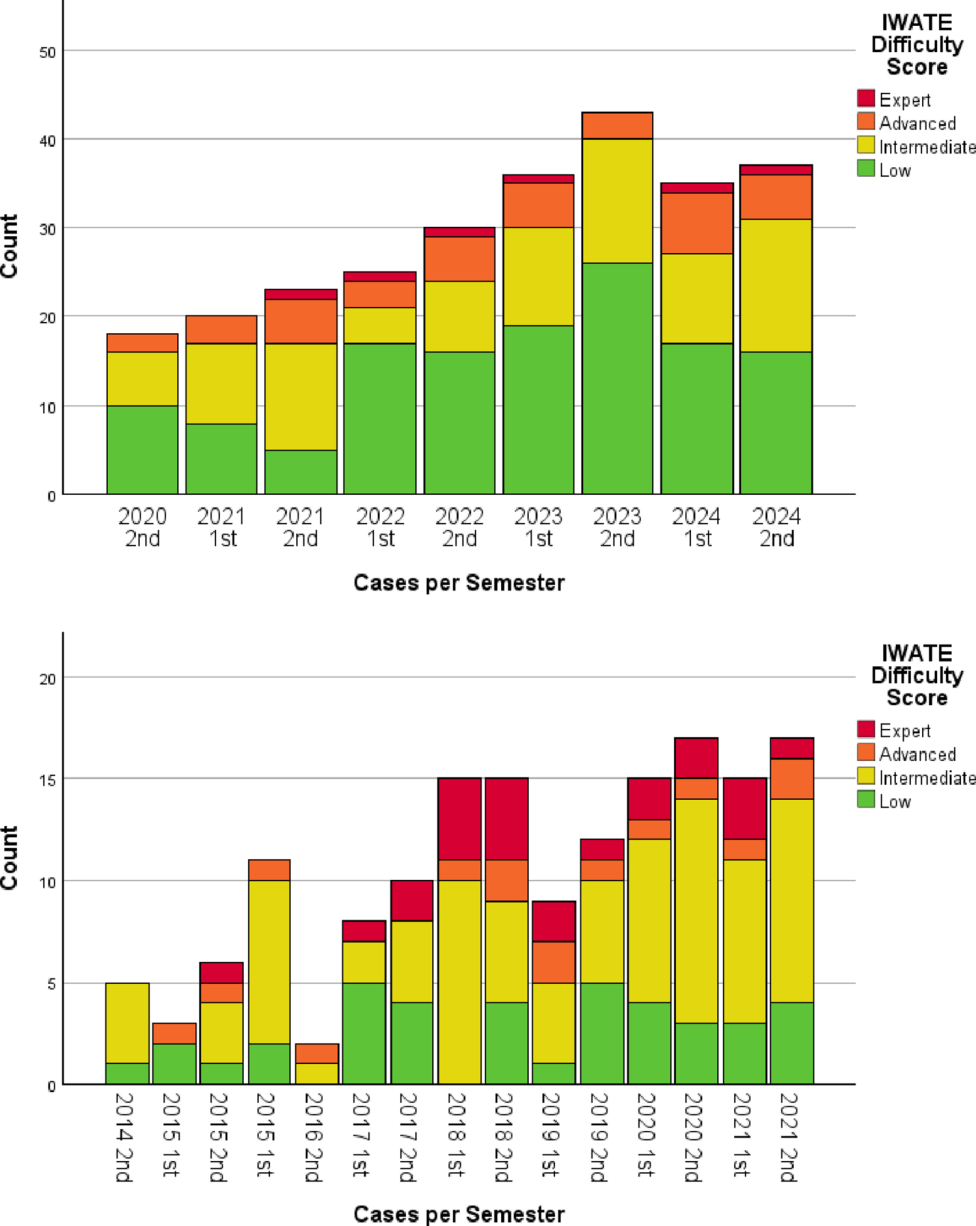
**a** Stacked bar chart of cases per semester at KUH – stratified by IWATE difficulty score. **b** Stacked bar chart of cases per semester at UMCU – stratified by IWATE difficulty score

A stacked bar graph with frequency of procedures per semester and the proportion of the different IWATE difficulty score levels is shown in Fig. 1a and b for KUH and UMCU, respectively. In both figures a trend to increasing volume is seen, more so at KUH than at UMCU. UMCU shows a preference towards increasing the complexity of cases before increasing the volume, while the opposite is true of KUH.

Tables 3 and 4 provide an overview of the surgeons introduced as console surgeon in each centre. In both hospitals a core team of three surgeons account for the majority of cases as console surgeon, with stronger concentration at UMCU (90.8%) than at KUH (71.1%). The spread of both the role as console surgeon and tableside surgeon was broader at KUH than at UMCU. The moving average analysis shows that the proficiency threshold of < 10% conversion rate and < 10% severe complication rate in a block of 20 cases is reached at case 124 at UMCU and case 133 at KUH.

**Table 3:** Console and tableside surgeons at Karolinska University Hospital

Surgeon	Console *N* (%)	Tableside *N* (%)
A	82(30.7)	46(17.2)
B	58(21.7)	31(11.6)
C	50(18.7)	26(9.7)
D	8(3.0)	9(3.3)
E	11(4.1)	6(2.2)
F	22(8.2)	16(6.0)
G	17(6.4)	41(15.4)
H	19(7.1)	38(14.2)
Other	0	54(20.2)

Surgeons listed in order of introduction. Category ‘Other’ is a range of tableside surgeons that were never in the console (Residents/Fellows/Visiting surgeons) with maximum 4 times as tableside surgeon

**Table 4:** Console and tableside surgeons at University Medical Center Utrecht

Surgeon	Console *N* (%)	Tableside *N* (%)
A	93(56.7)	30(18.3)
B	23(14.0)	35(21.1)
C	33(20.1)	27(16.5)
Other	15(9.1)	72(42.1)

Surgeons listed in order of introductions. Category ‘Other’ is a range of tableside surgeons that were up to 5 times in the console (Residents/Fellows/Visiting surgeons) with a maximum of 4 times as tableside surgeon

### Regression analysis

Binary logistic regression showed significant correlation between chronological case number and risk of the occurrence of early complications (OR=0.988, 95%CI [0.976-0.999], *p *= .034), indicating a decrease in odds of complications with center experience, independent of individual surgeon differences (*p* = .979). The interaction factor was added to assess individual learning curve differences (case number * surgeon) which showed no significant differences in learning progression (*p*=.952). A higher IWATE classification tends to lead to higher odds of complication; however, this result was not significant (OR = 1.216, 95%CI [0.893-1.658], *p *= .215).

In multivariate linear regression analyses, estimated blood loss and operative time were independently associated with factors related to operative complexity and intra-operative parameters. Estimated blood loss was associated with a small, but significant, reduction as case numbers progressed (β = -0.55, [-0.925–0.168], *p *= .005). Increasing IWATE difficulty score was associated with significantly increased blood loss (β = 31.38, [3.787–58.969], *p*=.026). Conversion to open surgery was associated with a substantial increase in blood loss (β = 322.75, [232.844-412.647], p = < 0.001) and operative time (β = 59.94, [11.031-108.853], *p*<.017).

After adjustment in linear regression, estimated blood loss and operative time were not significantly associated to hospitals (*p *= .166 and *p *=.703, respectively). Usage of the Pringle maneuver was strongly associated with a reduction in blood loss (β=-710.42, [-940.453—480.379], *p *< .001), while longer Pringle duration was independently associated with increased blood loss (β = 2.60, [0.840 − 4.350], *p *= .004) and longer operative time (β = 1.79, [0.944-2.635], *p *< .001). No independent associations were observed for console surgeon, ASA score, age, sex, history of abdominal surgery or cirrhosis and number of lesions. Longer operative time was independently associated with increased blood loss (β = 0.49, [0.214-0.772], *p *< .001), and greater blood loss was in turn independently associated with longer operative time (β = 0.12, [0.052-0.187], *p *< .001).

## Discussion

Most prior studies focus on technical feasibility and peri-operative outcomes, but few have addressed structured implementation. The optimal strategy for initiating robotic liver surgery programs in high-volume tertiary HPB centers remains unclear [2, 5, 32, 33]. This gap contributes to heterogeneity and may delay implementation, depriving patients of potential improvement in outcome [15, 25, 33–38].

Despite advances, robot-assisted surgery is not used in the majority of HPB procedures internationally [1]. Robotic liver surgery underwent a rapid rise from 10% to 25% of liver resections in the Netherlands in the period from 2020 to 2023 being performed robotically, but approximately 50% of liver surgery nationwide is still performed open [39].

This bi-institutional natural experiment observed that institutional proficiency in robotic liver surgery can be achieved through distinct implementation strategies, provided the introduction is intentionally structured from the outset. Despite differing approaches with a more early-adopter expertise-based focus at UMCU compared to the guided adoption and aim for broad training opportunities at KUH, both centers reached the proficiency threshold at comparable case volumes (124 and 133 cases, respectively).

The observed differences in implementation pathways reflect deliberate institutional strategies. During introduction, UMCU adopted a strategy to achieve their goals with a small and dedicated team, aimed at increasing complexity and extending indications for robotic approach in HPB surgery [2]. Prior to the introduction, UMCU surgeons followed Da Vinci console training and spent time observing robotic surgery in a high-volume center that had already started with robot-assisted HPB surgery. While surgeons were trained laparoscopically in general surgery, experience with laparoscopic liver surgery was limited to infrequent minor resections [40].

Contrastingly, KUH focused on increasing volume and training a broad spectrum of surgeons to operate robotically prioritizing exposure over early complex procedures [15]. Despite broader surgeon participation at KUH, adverse outcomes were not attributable to individual surgeons, instead this effect came from case complexity and improved with institutional experience, defined by chronological institutional case number. In the final year of the cohort, KUH performed 71 out of 252 (28%) liver surgeries robotically, slightly more than 32 out of 145 (24%) at UMCU, possibly a reflection of the increased operative time required for more complex procedures. The initially introduced KUH surgeons took at least 30 h of simulator training, followed by the Surgeon-Led Procedure Training Series (TR 300, Intuitive Surgical) at the Orsi Academy (Melle, Belgium). This was followed by case observations at UMCU and later with multiple rounds of on-site proctoring at KUH. Laparoscopic liver surgery experience was comparable to that of the UMCU surgeons.

Existing literature on robotic liver surgery learning curves has predominantly focused on individual surgeon proficiency, mostly reporting substantially lower-case numbers required to achieve proficiency and/or mastery [24, 26, 28, 41–43]. Such estimates may not reflect the institutional learning process in high-volume HPB centers introducing robotic liver surgery, where multiple surgeons are trained concurrently and case-complexity and volume evolve over time.

Comparative parameters are required to define institutional proficiency and successful implementation. In the current literature on proficiency and implementation of robotic liver surgery the most frequently used parameters are conversion rate, severe complication rate, estimated blood loss and operative time [23, 25–28, 31, 41, 42, 44]. Severe complications and conversion are binary and relatively frequently occurring events in hepatobiliary surgery, with benchmarks reported around 10%, and lower for the mastery phase [23, 27, 28, 43]. For these parameters, reaching stability below the benchmark not only demonstrates an individual surgeon’s proficiency of procedures, but also manifests proper patient selection, good team dynamics and lack of intra-operative technical limitations.

Estimated blood loss and operative time are continuous parameters that may show a decrease as experience grows. However, this effect will be severely confounded by case-mix, difficulty level of the procedures performed and the introduction of new surgeons to robot-assisted surgery, negating institutional improvement effects [23, 26, 42]. These factors, combined with possible subjectivity and variance in institutional reporting may lead to less reliable comparisons, but due to known clinical relevance and influence on operative outcomes were chosen as secondary outcomes [45, 46].

By pre-defining proficiency at the institutional level, including case-mix adjustments using the IWATE difficulty score, this study presents a realistic example of institutional implementation pathways. To the authors’ knowledge, no research comparing institutional implementation pathways and development strategies like this has been published.

From a clinical perspective, these findings highlight the importance of defining a clear implementation strategy before initiating a robotic liver surgery program in an institution. Centers aiming to function as a regional/national training hub may benefit from more restrictive case selection with structured proctor guidance, while centers pursuing technical stewardship may prioritize limiting team size in order to escalate complexity faster. Both approaches require clearly pre-defined agreements, both within the surgical team and at an institutional level, allowing for adequate access to robotic systems so as to be minimally restricted by case scheduling and training opportunities. The decision to pursue either strategy must be ethically weighed according to the local caseload, regional experience and availability and responsibilities of the surgeons to be trained. Based on this research, it cannot be stated that one of the strategies is superior, allowing both options to be feasible, within local center constrains. While the centralized nature of HPB surgery in the Dutch and Swedish healthcare systems may limit generalizability to all centers, the underlying principles and pathways are relevant to other high-volume centers. Though introduction of robotic liver surgery took place at different timepoints, no fundamental changes in the surgical management of these patients took place in the same period.

This study has several strengths, including the bi-institutional design, large cohort of patients and use of validated difficulty scoring methods to adjust for case complexity. The choice for the proficiency thresholds reflects clinically relevant outcomes on both institutional and individual patient level, however does not reflect long-term oncological outcomes. The retrospective nature of part of the data collected is a limitation. Selection bias may be present, but it was the clear aim to increase not only in volume of robot-assisted hepatobiliary surgery, but also in proportion of the total performed liver surgeries. To avoid sampling bias, all patients were discussed in the multidisciplinary conferences in respective centers where course of treatment, including surgical plan, was decided with all involved treatment providers. Heterogeneity among local practices is not avoidable, but with analysis split by center, was equally applied to all patients in respective cohorts. Known confounders were included in the regression analysis, but hidden confounders might remain.

In conclusion, structured implementation allows for safe adoption of robotic liver surgery in high-volume HPB centers, regardless of a strategic focus on team expansion or increasing complexity. Early definition of the institutional goals and alignment of training pathways with these objectives is essential for efficient and sustainable program development.

## Data Availability

No datasets were generated or analysed during the current study.
